# Impact of bauxite mining on mosquito communities (Diptera: Culicidae) and pathogen transmission risks in the eastern Amazon

**DOI:** 10.1111/mve.70107

**Published:** 2026-08-24

**Authors:** Mayron Mielly Morais Pacheco, Poliana da Silva Lemos, Mônica Silva Coelho, Tatiane Marques Porangaba de Oliveira, Luciano Chaves Franco Filho, Øystein Wiig, Ana Cristina Mendes‐Oliveira, Leonardo Sena, Maria Anice Mureb Sallum

**Affiliations:** ^1^ University of São Paulo, School of Public Health São Paulo São Paulo Brazil; ^2^ Federal University of Pará Belém Pará Brazil; ^3^ Department of Epidemiology University of São Paulo, School of Public Health São Paulo São Paulo Brazil; ^4^ Evandro Chagas Institute Ananindeua Pará Brazil; ^5^ Natural History Museum, University of Oslo Oslo Norway; ^6^ Institute of Biological Sciences Federal University of Pará Belém Pará Brazil

**Keywords:** Amazon, Blood‐feeding, Diversity, Mining, Spillover, Vectors

## Abstract

Mining activities pose significant and often underestimated threats to tropical forests, with distinct ecological implications for various taxa, including mosquitoes. This study aimed to investigate the effects of bauxite mining on the diversity, composition and blood‐feeding habits of mosquito communities in the Eastern Amazon. The research was conducted in the operational area of Mineração Paragominas S.A., located in the state of Pará, Brazil, where mosquitoes were collected using CDC light traps and active search methods. Species‐level taxonomic identification was performed using morphological keys and molecular techniques. Blood meal sources were identified by targeting three mitochondrial genes: 16S, COI and Cytb. Diversity analyses were based on Rényi entropy and rarefaction and extrapolation curves, while compositional changes were assessed through Principal Coordinates Analysis (PCoA). Results showed that bauxite mining significantly alters mosquito community structure in the Eastern Amazon, with critical public health implications. Three main ecological patterns were identified: (1) increased abundance of vector and anthropophilic species (e.g., *Aedes albopictus* and *Culex quinquefasciatus*) in mining areas, demonstrating their capacity to invade, establish and proliferate in remote forest habitats; (2) shifts in community composition, with the replacement of forest‐dwelling species by generalists in degraded areas and (3) a reduction in the richness of blood sources in impacted areas, compromising the protective dilution effect typically observed in preserved ecosystems. These findings identify mining zones as hotspots for spillover and spillback events, underscoring the need for integrated monitoring (vector–pathogen–host) and artificial breeding site control to mitigate public health risks.

## Graphical abstract

- Bauxite mining in the Amazon alters mosquito communities, favouring anthropophilic and generalist vectors while reducing species diversity and the variety of blood meal sources.
- Molecular analyses identified humans, tapirs and deer as the primary blood meal sources. The lower vertebrate richness in mining areas intensifies the risk of pathogen transmission due to the loss of the dilution effect.
- Mining areas represent potential hotspots for spillover and spillback events. An integrated monitoring approach (vectors‐pathogens‐hosts) is essential to mitigate threats to public health.

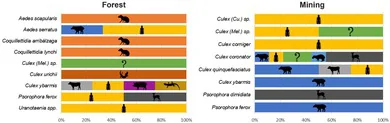

## INTRODUCTION

Mosquitoes (Diptera: Culicidae), with 3727 formally described species, are among the deadliest creatures on Earth, acting as vectors for protozoa (e.g., *Plasmodium* spp.), filarial worms (e.g., *Wuchereria bancrofti*) and over a hundred viruses, including dengue virus (*Orthoflavivirus denguei*—DENV), chikungunya virus (*Alphavirus chikungunya*—CHIKV) and Zika virus (*Orthoflavivirus zikaense*—ZIKV) (Girard et al., 2020; Harbach, 2024). In 2024, it was estimated that more than 12 million dengue cases and approximately 8000 related deaths occurred in the Americas, with Brazil accounting for nearly 80% of the cases (PAHO, 2025). Globally, malaria caused over 263 million cases and 577,000 deaths in 83 countries in 2023, including 140,265 infections and 63 deaths reported in Brazil (Brazil, 2022; WHO, 2024). These epidemiological data underscore the need to understand the ecological factors influencing mosquito population dynamics and their role in the emergence of transmissible diseases, particularly in high‐biodiversity regions such as the Amazon (Cincotta et al., 2000; Fearnside, 2021).

The Amazon Rainforest, one of the most biodiverse biomes on Earth, has undergone rapid transformation due to anthropogenic activities. The so‐called ‘arc of deforestation’, which encompasses the southern and eastern edges of the Brazilian Amazon, accounts for about 80% of regional forest loss (Bebbington et al., 2018; Fearnside et al., 2009). In this region, mining, large‐scale agriculture and extensive cattle ranching have led to various environmental impacts (Fearnside et al., 2009; Murguía et al., 2016; Sonter et al., 2017).

Among these activities, mining in tropical forests may be responsible for complete landscape alteration due to vegetation removal, soil stripping, environmental contamination, biodiversity loss, habitat fragmentation and increased edge effects (Kahhat et al., 2019; Murguía et al., 2016; Sonter et al., 2017). Furthermore, reforestation through Degraded Area Recovery Plans (PRADs) in mined regions can introduce additional impacts, as the resulting ecological succession alters environmental conditions and consequently affects the abundance, composition and distribution of various species (Cerqueira et al., 2021; De Oliveira Brito et al., 2024).

Although recent studies have assessed the impact of edge effects across landscape gradients on mosquito communities (Almeida et al., 2023; Hendy et al., 2020, 2023, 2025; Meyer Steiger et al., 2016; Young et al., 2021), the dynamics of these communities in mining areas remain underexplored. This knowledge gap is particularly relevant because mining areas create highly contrasting anthropogenic matrices, where edge effects may be more intense (Ewers & Didham, 2007; Guerra et al., 2017). Any shifts in mosquito diversity and community composition, or in blood‐feeding patterns induced by the harshness of these landscapes, can lead to the exploitation of new hosts and increased contact with diverse vertebrates, thereby heightening the risk of pathogen transmission, including spillover and spillback events (Burkett‐Cadena & Vittor, 2018; Hendy et al., 2025; Pereira‐dos‐Santos et al., 2020).

According to Fikrig and Harrington (2021) and Hendy et al. (2025), accurately understanding how forest edge landscapes influence mosquito community structure and blood‐feeding behaviour enables researchers to investigate changes in diversity and composition, host adaptation, vector competence, transmission dynamics and the identification of potential reservoirs, ultimately aiding in reducing human‐mosquito contact. Therefore, environmental transformations caused by mining may significantly affect mosquito populations, offering crucial insights for anticipating and mitigating public health risks. Within this context, the present study aims to investigate the effects of bauxite mining on the diversity, composition and blood‐feeding habits of mosquito communities in the Eastern Amazon.

## MATERIALS AND METHODS

### Ethics statement

Mosquito collections were authorized by SISBIO (permit no. 69667‐1), as part of the project ‘Metagenomics and metabarcoding as tools for assessing ecosystem health in mining areas of Hydro Paragominas—PA’ (Hydro/UFPA/Fadesp 4180).

### Study area

The study was conducted in a bauxite mining area belonging to Mineração Paragominas S.A. (MPSA), part of the Hydro Group (Norsk Hydro ASA), located in the municipality of Paragominas, state of Pará, Brazil.

The region's climate (Köppen–Geiger classification: Awi) comprises four distinct periods: rainy (February–May), dry spell (June), dry season (July–November) and transition (December–January), with an average annual temperature of 26.3°C, relative humidity of 81% and precipitation of 1742.9 mm (Bastos et al., 2005). The original vegetation is submontane dense ombrophilous forest, with areas at different stages of degradation (IBGE, 2012).

Five sampling units were distributed across two main areas, Forest and Mining, with distinct landscape features (Figure 1 and Table 1). In the Forest area, two units were established: ‘Continuous Forest’ (control unit), located in a Permanent Preservation Area (PPA) and ‘Roadside’, situated in a forest‐road edge environment with regular traffic of both light and heavy vehicles. In the Mining area, three edge units were established: ‘Office’, near residential and administrative buildings with high human activity; ‘Stations’, adjacent to an operational mining complex and ‘Pasture’, in a grazing area undergoing early forest recovery under a Degraded Area Recovery Plan (PRAD).

**FIGURE 1:**
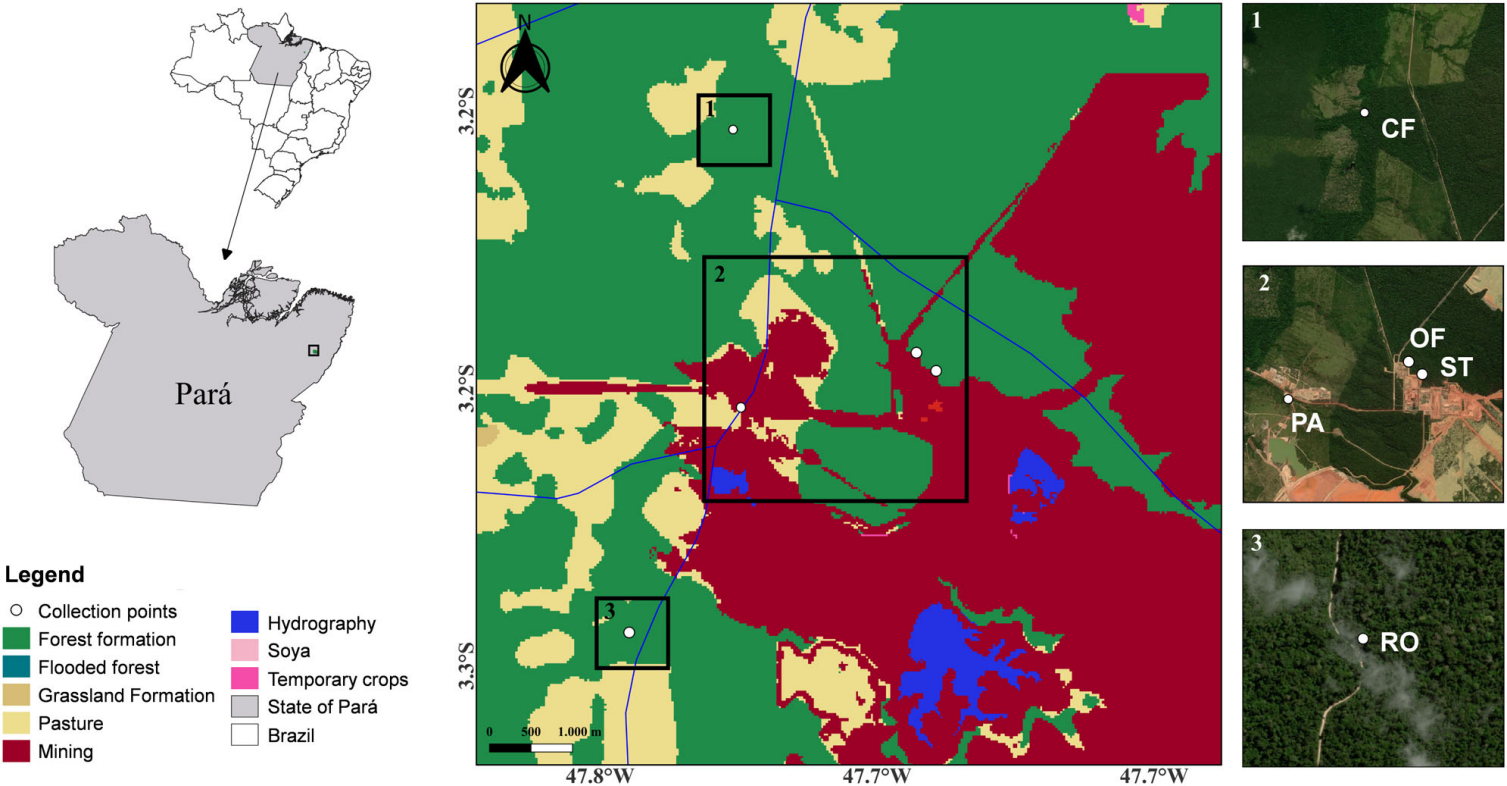
Map of the study area showing sampling units and land use within the Mineração Paragominas S.A. (MPSA) property, located in Paragominas municipality, Pará, Brazil. Different land use types are represented by distinct colours, based on the most recent MapBiomas land cover data (MapBiomas Project, 2024), with a spatial resolution of 30 m. Sampling units are indicated by dots (●) and abbreviations: CF, Continuous Forest; OF, Office; ST, Stations; PA, Pasture; RO, Roadside. *Source*: Mapbiomas, 2024.

**TABLE 1:** Geographic, landscape, and sampling characteristics of sampling units located in the Mineração Paragominas S.A. (MPSA) area, Paragominas, PA, from July 2022 to May 2023.

Collection points	Environment	Geographic coordinates	Canopy	Vegetation types
Continuous forest	Unfragmented forest	−3.2220, −47.7556	+20 m	Slightly degraded submontane forests
Roadside	Road‐forest edge	−3.2764, −47.7668	+20 m	Degraded submontane forests
Office	Mining‐forest edge	−3.2464, −47.7354	+20 m	Degraded submontane forests
Stations	Mining‐forest edge	−3.2481, −47.7336	+20 m	Degraded submontane forests
Pasture	Pasture‐forest edge	−3.2520, −47.7547	~5 m	Forest recovering after mining

### Mosquito collection and morphological identification

Culicids were sampled bimonthly over 1 year, from July 2022 to May 2023, covering both rainy and dry seasons. During each of the six sampling campaigns, mosquitoes were collected using active search (2 h/unit) during daylight hours with an entomological net and manual aspirator, avoiding direct contact with the operator (Consoli & Oliveira, 1994). At night, one CDC light trap (Centers for Disease Control) was installed per unit, equipped with white light bulbs, placed 1.5 meters above ground and operated for 12 h (6:00 p.m.–6:00 a.m.).

Specimens were preserved in liquid nitrogen and later morphologically identified using taxonomic keys by Consoli and Oliveira (1994); Forattini (2002) and Sallum et al. (2020). For Anophelinae, we adopted the nomenclature proposed by Foster et al. (2017). All females with visible blood meals or blood traces in the abdomen were individually stored in 1.5 mL plastic tubes.

Some male mosquitoes were selected for genitalia‐based morphological identification using keys by Berlin and Belkin (1980); Forattini (2002); Rozeboom and Komp (1950) and Valencia (1973). Dissected specimens mounted in Canada balsam on microscope slides (Forattini, 1962) were deposited in the Entomological Reference Collection of the School of Public Health, University of São Paulo (FSP/USP), under accession numbers E‐16521 to E‐16528. These reference males were used to amplify a fragment of the D2 domain of the 28S large subunit ribosomal RNA gene for the species identification of blood‐fed females that could not be morphologically identified. The D2 was chosen instead of the barcode region of the mitochondrial cytochrome c oxidase subunit I (*COI*) gene because of contamination of mosquito DNA with vertebrate DNA in blood‐fed females.

### Blood meal identification

DNA extraction and purification were performed using the PureLink™ Genomic DNA Mini Kit (Thermo Fisher Scientific, USA), following the manufacturer's Blood Spots protocol, using entire blood‐fed mosquitoes (head, thorax and abdomen). Blood meal sources were identified by amplifying fragments of three mitochondrial genes: cytochrome b oxidase (*Cytb*), cytochrome c oxidase I (*COI*) and 16S rRNA (16S), using the primers listed in Table 2. Thermocycling conditions followed Schnell et al. (2018) for 16S and Townzen et al. (2008) for *COI* and *Cytb*. Polymerase Chain Reaction (PCR) products were visualised on 2% agarose gels and purified using a PEG/NaCl precipitation protocol (20% polyethylene glycol 8000/2.5 M NaCl), then sequenced via Sanger sequencing (Microsynth Seqlab GmbH, Göttingen, Germany). All experimental steps included negative controls to ensure reliability.

**TABLE 2:** Primer sequences used to amplify fragments of three mitochondrial genes: 16S, *COI*, and *Cytb*.

Primers	Sequences (5′ a 3′)	References
*16S*‐fw	CGGTTGGGGTGACCTCGGA	Taylor (1996)
*16S*‐rv	GCTGTTATCCCTAGGGTAACT	
*COI*‐fw	GCAGGAACAGGWTGAACCG	Townzen et al. (2008)
*COI*‐rv	AAGAATCAGAATARGTGTTG	
*Cytb*‐fw	GAGGMCAAATATCATTCTGAGG	
*Cytb*‐rv	TAGGGCVAGGACTCCTCCTAGT	

Abbreviations: Fw, forward; rv, reverse.

Sequences were edited in BioEdit 7.2.5 (Hall, 1999), with primers trimmed, and compared against the NCBI database using BLAST (minimum similarity ≥98%). The online CIRCOS tool (http://circos.ca/) was used to graphically represent the relative abundance and distribution of blood sources by mosquito species (Krzywinski et al., 2009).

### Molecular identification of mosquitoes

DNA from morphologically identified male specimens was extracted using the salting‐out method (Miller et al., 1988). Blood‐fed females were identified by matching their nucleotide sequences to males using a fragment of the D2 domain of the 28S large subunit ribosomal RNA gene. PCR amplification was performed in a final volume of 20 μL containing 1 × GoTaq® Master Mix (Promega Corporation, Madison, USA), 0.2 μM of each primer (Ill+Mozzie.D2.Uni.F TCGTCGCAGCGTCAGATGTGTATAAGAGACAGAAGCACTCTGAATAGAGAGTC and Ill+Mozzie.D2.Uni.R GTCTCGTGGGCTCGGAGATGTGTATAAGAGACAGTGGTCCGTGTTTCAAGAC), 2–5 ng of DNAand ultrapure water (Pedro et al., 2020). The thermocycling conditions consisted of 95°C for 2 min, 30 cycles of 95°C for 10 s, 65°C for 30 s, 72°C for 30 s and 72°C for 5 min as final extension.

PCR products were visualised on 1.5% agarose gels. Purification, sequencing and editing followed the same protocol used for blood meal identification. Sequence similarity ≥98% with male reference sequences (via BLAST) was used to assign females to species.

### Statistical analyses

Species richness and diversity across areas and sampling units were assessed using individual‐based rarefaction and extrapolation curves. Hill numbers were calculated for *q* = 0 (species richness), *q* = 1 (Shannon diversity) and *q* = 2 (Simpson diversity), as described by Chao et al. (2014). Confidence intervals (95%) were estimated using bootstrap (999 replicates) as per Hsieh et al. (2016). Analyses were conducted using the iNEXT package in RStudio (Hsieh et al., 2016).

Rényi entropy (H*α*) was used to evaluate diversity across environments, offering multiple perspectives on community structure (Rényi, 1961; Tóthmérész, 1995). This index varies with the parameter *α*: *α* = 0 reflects species count; *α* = 1 corresponds to the Shannon index and *α* = 2, to the Simpson index (Ricotta, 2003; Ricotta & Avena, 2002). This approach allows for community ordering and the detection of diversity intersections (Ricotta, 2003).

Principal Coordinates Analysis (PCoA) was used to ordinate samples by species composition, based on the Bray‐Curtis index applied to log‐transformed abundance data to minimise outlier influence (Fukuyama et al., 2012). A Venn diagram illustrated species overlap and uniqueness among sample groups (Shade & Handelsman, 2012). All analyses were performed in RStudio v4.4.2 using the vegan package (Oksanen, 2015).

## RESULTS

### Overall abundance and species diversity

A total of 2458 mosquitoes were collected, with a female‐to‐male ratio of 2.74 and only 0.12% of individuals not sexed. The taxonomic composition included 16 genera and 35 species, with *Culex* and *Aedes* being the most diverse genera (seven species each), followed by *Psorophora* (five species) (Table 3). However, 69.2% of the specimens (*n* = 1701) could not be identified to the species level due to morphological damage and were categorised into higher or informal taxonomic groups (e.g., groups, sections, complexes) (Table 3).

Among the identified species, 11 were classified as abundant (≥10 individuals), representing 90.48% of the specimens identified to the species level. *Culex quinquefasciatus* was the dominant species (*n* = 371), followed by *Aedes serratus* (*n* = 90) and *Aedes scapularis* (*n* = 41). In contrast, 24 species were considered rare (<10 individuals), 10 of which were represented by a single specimen (Table 3).

CDC light traps accounted for 65.46% of the total captures, being most effective for *Uranotaenia calosomata* (1.06%), *Ae. serratus* (0.99%) and *Psorophora dimidiata* (0.87%). In contrast, active search methods (34.54% of captures) were more effective for *Cx. quinquefasciatus* (43.75%), *Ae. serratus* (8.73%) and *Ae. scapularis* (4.01%), highlighting differences in attractiveness between methods (Table 3).

**TABLE 3:** Species composition of mosquitoes collected in the Mineração Paragominas S.A. (MPSA) area, Paragominas, Pará, from July 2022 to May 2023.

Genus	*N* (%)	Forest		Mining			Sex	Collection		Seasonality	
Subgenus											
Specie authority, date		CF	RO	OF	ST	PA		AS	CDC	Rainy	Dry
Subfamily Anophelinae											
Tribe Anophelini											
*Anopheles*											
spp.	9 (0.37)	5	0	0	0	4	8F + 1 M	2	7	1	8
*Nyssorhynchus*											
spp.	61 (2.48)	22	0	15	17	7	61F		61	5	56
Total number of Anophelinae specimens	70	27	0	15	17	11	69F + 1 M	2	68	6	64
Subfamily Culicinae											
Tribe Aedeomyiini											
*Aedeomyia*											
(*Aedeomyia*)											
*squamipennis* (Lynch Arribálzaga, 1878)	1 (0.04)	0	0	0	0	1	1F		1	1	
Tribe Aedini											
*Aedes*											
(*Howardina*)											
*arborealis* Bonne‐Wepster & Bonne, 1920	22 (0.90)	1	16	1	4	0	20F + 1 M + 1 UN.	21	1	21	1
spp.	3 (0.12)	1	0	2	0	0	2F + 1 M	3		3	
(*Ochlerotatus*)											
*fulvus* (Wiedemann, 1828)	8 (0.33)	4	3	0	1	0	8F	4	4	6	2
*hastatus* Dyar, 1922	1 (0.04)	0	1	0	0	0	1F	1		1	
*scapularis* (Rondani, 1848)	41 (1.67)	10	21	6	0	4	29F + 12 M	34	7	10	31
*serratus* (Theobald, 1901)	90 (3.66)	24	25	11	13	17	90F	74	16	66	24
spp.	10 (0.41)	2	2	0	0	6	1F + 9 M	10		8	2
(*Protomacleaya*)											
*argyrothorax* Bonne‐Wepster & Bonne 1920	1 (0.04)	0	0	0	0	1	1F		1		1
(*Stegomyia*)											
*albopictus* (Skuse, 1895)	35 (1.42)	0	0	6	2	27	30F + 5 M	33	2	22	13
sp.	2 (0.08)	0	1	1	0	0	1F + 1 M	1	1	1	1
*Haemagogus*											
(*Conopostegus*)											
*leucocelaenus* (Dyar & Shannon, 1924)	1 (0.04)	0	0	0	1	0	1F	1			1
*Psorophora*											
(*Grabhamia*)											
*dimidiata* Cerqueira, 1943	19 (0.77)	5	0	3	8	3	19F	5	14	12	7
spp.	16 (0.65)	1	6	2	3	4	15F + 1 M	5	11	7	9
(*Janthinosoma*)											
*albipes* (Theobald, 1907)	4 (0.16)	1	0	0	2	1	2F + 2 M	4		1	3
*ferox* (Von Humboldt, 1819)	35 (1.42)	25	7	1	1	1	33F + 1 M + 1Fmol	22	13	8	27
*lutzii* (Theobald, 1901)	7 (0.28)	3	3	0	1	0	7F	4	3	6	1
spp.	15 (0.61)	0	12	0	3	0	14F + 1 M	15		14	1
(*Psorophora*)											
*cilipes* (Fabricius, 1805)	1 (0.04)	0	1	0	0	0	1F		1	1	
sp.	2 (0.08)	0	1	1	0	0	2 M	2		1	1
spp.	2 (0.08)	2	0	0	0	0	1F + 1 M	2			2
Tribe Culicini											
*Culex*											
(*Anoedioporpa*)											
*luteopleurus* (Theobald, 1903)	1 (0.04)	1	0	0	0	0	1 M	1		1	
(*Carrollia*)											
*urichii* (Coquillett, 1906)	4 (0.16)	1	2	1	0	0	3Mgen + 1Fmol	4		4	
spp.	2 (0.08)	1	1	0	0	0	2F	2			2
(*Culex*)											
Complexo Coronator	193 (7.85)	1	1	30	137	24	190F + 3 M	15	178	7	186
*coronator* Dyar & Snab, 1906	21 (0.85)	0	9	5	2	5	12 Mgen + 9Fmol	13	8	15	6
*mollis* Dyar & Snab, 1906	5 (0.20)	1	0	0	1	3	5Mgen	5		5	
*quinquefasciatus* Say,1823	371 (15.09)	0	0	3	0	368	39F + 332 M	371		218	153
spp.	918 (37.35)	23	34	177	537	147	790F + 128 M	47	871	328	590
(*Melanoconion*)											
*ybarmis* Dyar, 1920	7 (0.28)	4	0	0	1	2	1 Mgen + 6Fmol	5	2	3	4
spp.	237 (9.64)	90	30	19	42	56	202F + 35 M	49	188	59	178
(*Phenacomyia*)											
*corniger* Theobald, 1903	17 (0.69)	0	0	0	1	16	15F + 2 M	5	12	11	6
spp.	136 (5.53)	41	76	8	9	2	62F + 74 M	16	120	5	131
*Lutzia*											
*bigoti* Bellardi, 1862	2 (0.08)	0	0	0	0	2	2F		2		2
Tribe Mansoniini											
*Coquillettidia*											
(*Rhynchotaenia*)											
*arribalzagae* (Theobald, 1903)	2 (0.08)	2	0	0	0	0	1F + 1 M	2		2	
*lynch* (Shannon, 1931)	3 (0.12)	1	0	1	0	1	3F		3	1	2
*venezuelensis* (Theobald, 1912)	6 (0.24)	0	1	3	1	1	6F		6		6
spp.	5 (0.20)	0	0	0	2	3	5F + 2 UN.	3	2		5
sp.	1 (0.04)	1	0	0	0	0	1F	1		1	
*Mansonia*											
(*Mansonia*)											
*pseudotitillans* Theolbald, 1901	1 (0.04)	0	1	0	0	0	1F		1		1
spp.	5 (0.20)	3	0	2	0	0	3F + 2 M	2	3		5
Tribe Orthopodomyiini											
*Orthopodomyia*											
*fascipes* (Coquillet, 1906)	16 (0.65)	9	6	1	0	0	7F + 9 M	16			16
sp.	1 (0.04)	0	0	0	1	0	1 M	1		1	
Tribe Sabethini											
*Limatus*											
*durhamii* Theobald, 1901	3 (0.12)	1	0	1	0	1	3F	3		3	
spp.	20 (0.81)	5	8	2	2	3	18F + 2 M	18	2	7	13
*Sabethes*											
(*Sabethes*)											
*glaucodaemon* (Day & Shannon, 1925)	1 (0.04)	0	0	0	1	0	1F	1			1
*quasicyaneus* Peryassú, 1922	6 (0.26)	2	0	3	1	0	6F	6		1	5
*Trichoprosopon*											
*digitatum* (Rondani, 1848)	3 (0.12)	1	1	1	0	0	1F + 2 M	3		3	
*Wyeomyia*											
(*Tryamyia*)											
*aporonoma* Dyar & Knab, 1906	2 (0.08)	0	0	0	2	0	2F	2			2
spp.	14 (0.57)	4	2	3	5	0	14F	14		14	
Tribe Uranotaeniini											
*Uranotaenia*											
(*Uranotaenia*)											
*calosomata* Dyar & Knab, 1907	17 (0.69)	9	7	1	0	0	15F + 2 M		17		17
*geometrica* Theobald, 1901	1 (0.04)	0	0	1	0	0	1F		1		1
*hystera* Dyar & Knab, 1913	1 (0.04)	0	0	1	0	0	1F		1		1
spp.	35 (1.42)	33	1	1	0	0	32F + 3 M		35	21	14
spp.	14 (0.57)	11	0	1	1	1	14F		14	5	9
Total number of Culicinae specimens	2387	324	279	299	785	700		846	1541	904	1483
Unidentified	1	0	0	0	1	0		1		1	
Total number of species	35	19	15	18	17	17		25	21	24	26
Total number of genera	16	13	10	12	11	10		12	11	13	14
Total number of specimens	2458	351	279	314	802	711		849	1609	911	1547

*Note*: Fmol: Molecularly identified; Mgen: Identified by male genitalia morphology.

Abbreviations: AS, active searching; CDC, Centers for Disease Control; CF, Continuous Forest; OF, Office; PA, Pasture; RO, Roadside; ST, Stations; UN., Unidentified.

The dry season accounted for 62.96% of total captures, with a peak in November (*n* = 759; 30.87%). The rainy season accounted for 37.04%, with the highest abundance in January (*n* = 497; 20.21%) (Figure 2, Table 3). Some species exhibited strong seasonal preferences: *Ur*. *calosomata* and *Orthopodomyia fascipes* were exclusive to the dry season, whereas *Cx*. *quinquefasciatus* and *Ae*. *serratus* maintained high populations in both seasons, suggesting greater adaptability. No abundant species were exclusive to the rainy season (Figure 3, Table 3).

**FIGURE 2:**
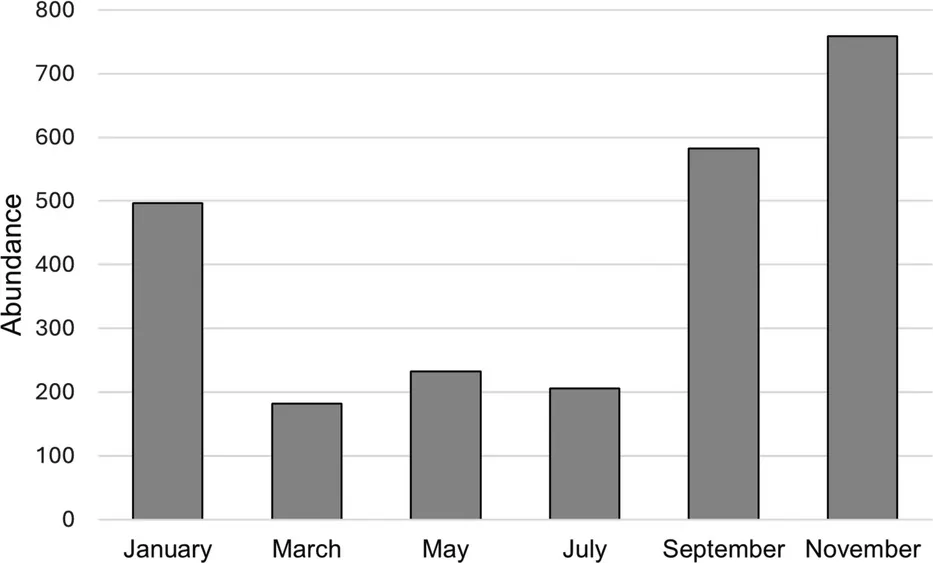
Temporal dynamics of mosquito specimens collected bimonthly during the dry (July–December) and rainy (January–June) seasons from July 2022 to May 2023 in the area of Mineração Paragominas S.A. (MPSA), Paragominas, PA.

**FIGURE 3:**
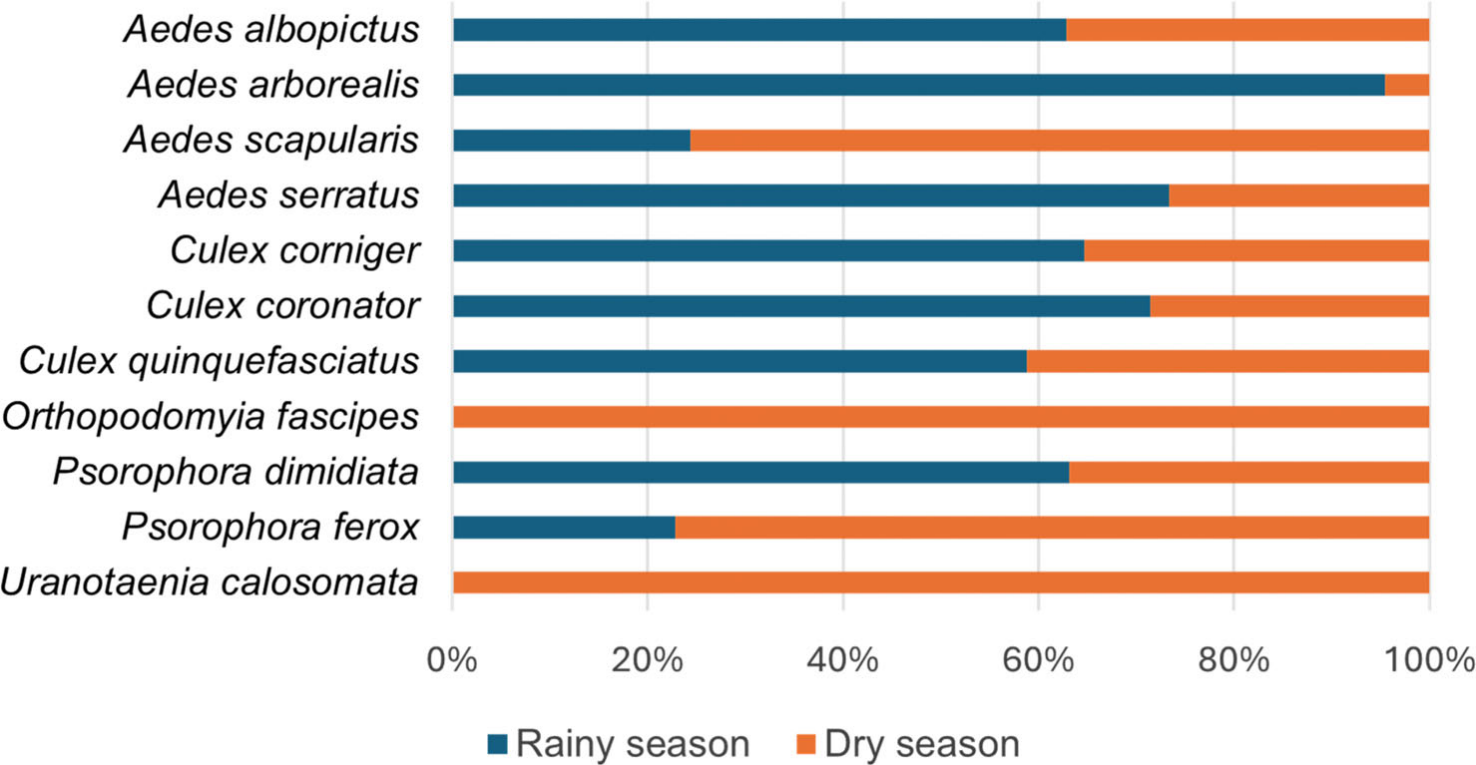
Relative seasonal frequency of the most abundant mosquito species collected bimonthly during the dry and rainy seasons, from July 2022 to May 2023, in the area of Mineração Paragominas S.A. (MPSA), Paragominas, PA.

### Differences in culicid fauna across mining landscapes

In the Mining area, 1828 specimens were collected, belonging to 16 genera and 30 species. The genera *Culex* (*n* = 1596), *Aedes* (*n* = 102), *Nyssorhynchus* (*n* = 39) and *Psorophora* (*n* = 34) together accounted for 96.9% of the collected specimens. Seven species were classified as abundant and 23 as rare, with 12 represented by a single specimen. The most abundant species were *Cx*. *quinquefasciatus* (20.31%), *Ae*. *serratus* (2.24%), *Aedes albopictus* (1.92%), *Cx. corniger* (0.93%), *Ps*. *dimidiata* (0.77%), *Cx. coronator* (0.65%) and *Ae*. *scapularis* (0.55%) (Figure 4).

In the Forest area, 630 specimens were collected, representing 13 genera and 24 species. The most represented genera were *Culex* (*n* = 316), *Aedes* (*n* = 111), *Psorophora* (*n* = 67) and *Uranotaenia* (*n* = 61), accounting for 88.09% of total specimens. Six species were considered abundant and 18 rare, with nine represented by a single specimen. The most abundant species were *Ae*. *serratus* (7.78%), *Ae*. *scapularis* and *Psorophora ferox* (both 4.92%), *Ae. arborealis* (2.70%), *Ur*. *calosomata* (2.54%) and *Or*. *fascipes* (2.38%) (Figure 4).

**FIGURE 4:**
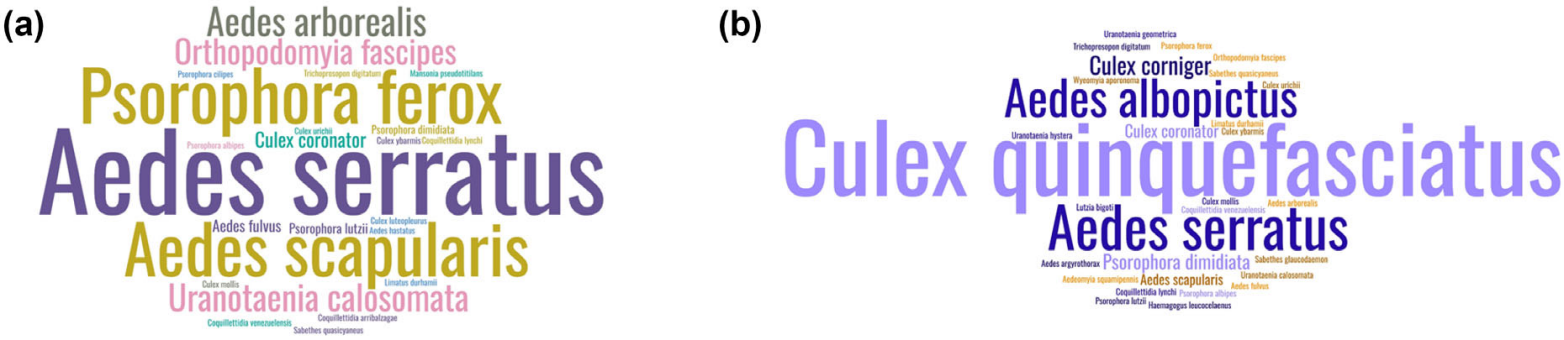
Word cloud of the relative frequency of culicid species collected in Forest (a) and Mining (b) areas within the Mineração Paragominas S.A. (MPSA), Paragominas, PA, from July 2022 to May 2023. Font size reflects species frequency.

Diversity analysis revealed that for *α* = 0 (sensitive to species richness), the Forest area showed lower diversity. However, as *α* increased, the Forest's Rényi entropy curve consistently surpassed that of the Mining area, indicating greater true diversity (*α* = 1) and more even species distribution (*α* = 2). Although the Mining area initially showed higher diversity values, its curve declined more steeply with increasing α, reflecting lower effective diversity and greater dominance of a few species (Figure 5).

**FIGURE 5:**
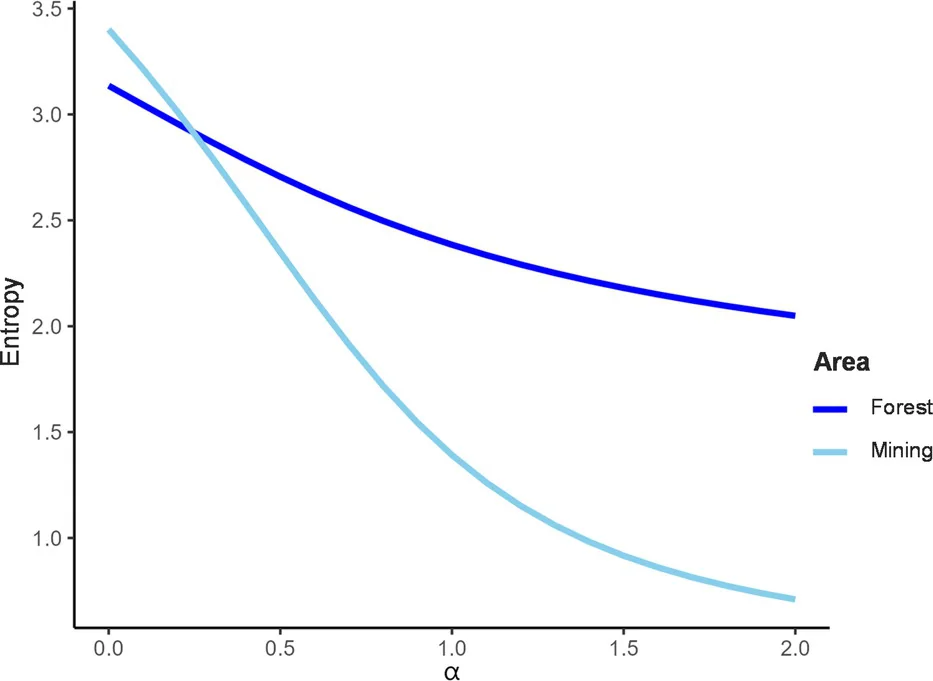
Rényi diversity curves showing differences between the Forest and Mining areas. *X*‐axis: *α* = 0 (species richness); *α* = 1 (Shannon–Weiner index); *α* = 2 (Simpson–Yule index); *α* = ∞ (Berger–Parker index).

Rarefaction and extrapolation curves reinforced these patterns, indicating that the Forest area exhibited greater interpolated and extrapolated species richness than the Mining area. Differences between environments were already evident in the interpolation stage, suggesting higher diversity in the Forest, even with fewer individuals sampled. Extrapolation showed that the Mining area's diversity reached saturation more quickly, while the Forest displayed greater potential for undiscovered species (Figure 6).

**FIGURE 6:**
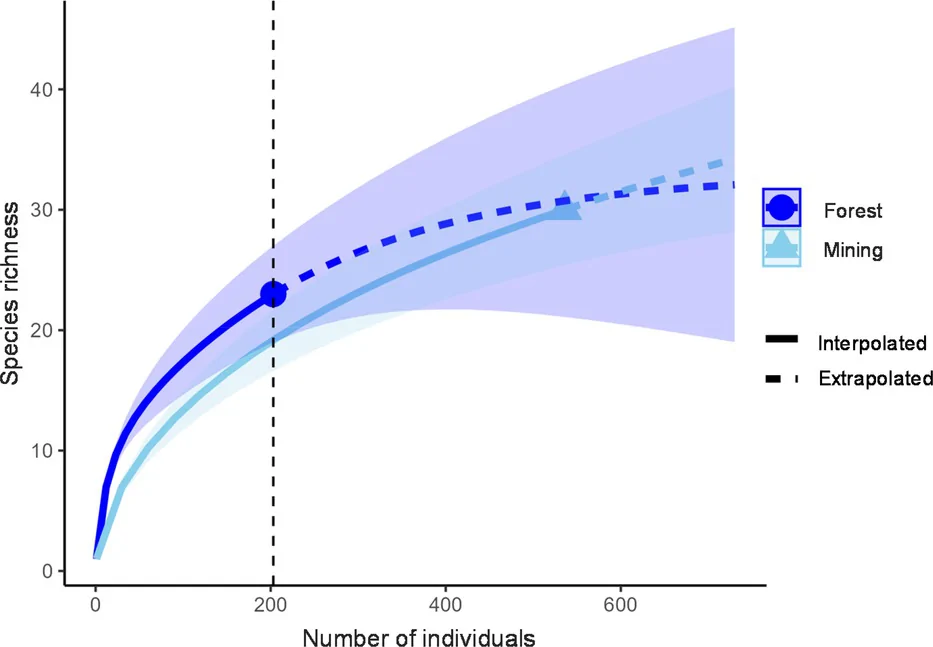
Interpolation and extrapolation curves based on the number of individual specimens found in the Forest and Mining areas. Shaded areas represent 95% confidence intervals.

PCoA showed that mosquito communities grouped into distinct ellipses, reflecting differences in composition and abundance distribution between environments. The Forest ellipse was more elongated, indicating greater species abundance dispersion, while the Mining ellipse was more compact, indicating lower variation. However, there was a notable overlap, especially along the PCoA1 axis, which explained 49.39% of total variation, suggesting the presence of generalist species capable of inhabiting both environments (Figure 7).

**FIGURE 7:**
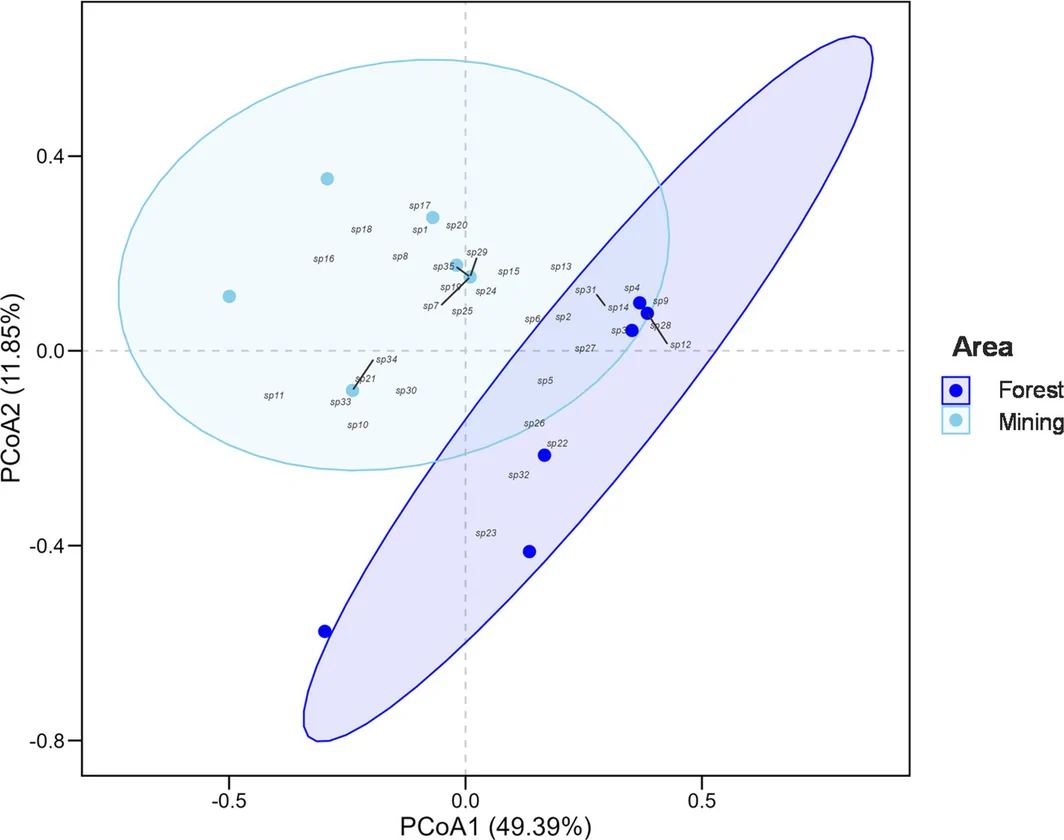
Principal Coordinates Analysis (PCoA) based on Bray–Curtis distances, comparing culicid communities between Forest and Mining areas.

### Habitat heterogeneity shapes mosquito communities

In the Continuous Forest unit, 351 specimens were collected, distributed across 13 genera and 19 species, dominated by *Culex* (46.44%), *Uranotaenia* (15.10%) and *Aedes* (11.96%). In the Roadside unit, 279 specimens were collected (10 genera, 15 species), with *Culex* (54.83%), *Aedes* (24.73%) and *Psorophora* (10.75%) being the most common (Figure 8). No anthropophilic species were recorded in either forest environment.

In the Office unit, 314 specimens were collected (12 genera, 18 species), dominated by *Culex* (77.38%), followed by *Aedes* (8.59%) and *Nyssorhynchus* (4.77%). The Stations unit yielded 802 specimens (11 genera, 17 species), with *Culex* (91.02%) as the dominant group, followed by *Aedes* (2.49%) and *Psorophora* (2.24%). In the Pasture unit, 711 specimens were recorded (10 genera, 17 species), mainly *Culex* (87.62%), *Aedes* (7.73%) and *Psorophora* (1.26%) (Figure 8). All three Mining‐associated sites (Office, Stations and Pasture) included anthropophilic species.

**FIGURE 8:**
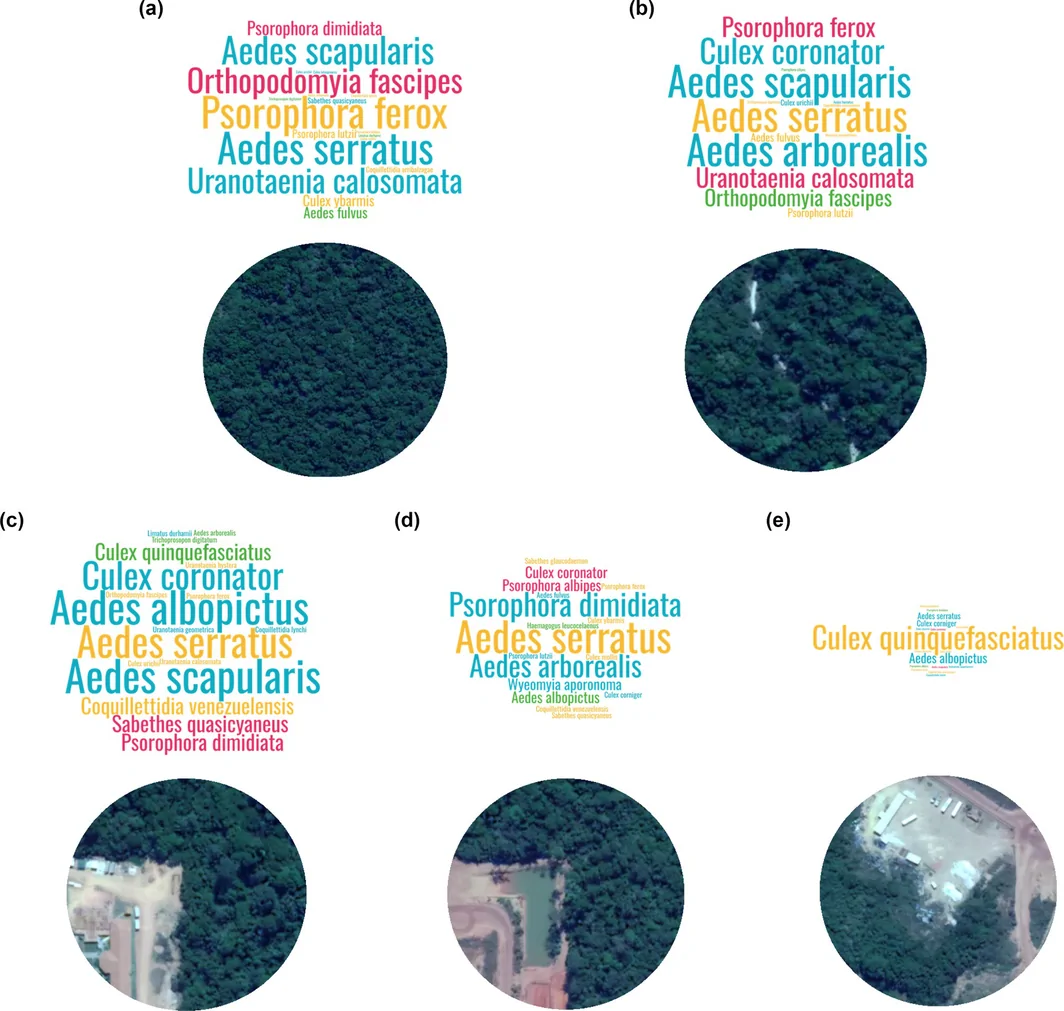
Word cloud of the relative frequency of culicid species collected in each sampling unit within Mineração Paragominas S.A. (MPSA), Paragominas, PA, from July 2022 to May 2023. (a) Continuous Forest; (b) Roadside; (c) Office; (d) Stations; (e) Pasture. Font size reflects species frequency.

Rényi diversity analysis showed that the Pasture had the steepest curve, reflecting low diversity (*α* = 1) and strong dominance by a few species (*α* = 2). In contrast, the Office unit showed the highest entropy values across all *α* levels, indicating greater diversity and a more even distribution. Continuous Forest, Roadside and Stations exhibited intermediate curves with gentler slopes, suggesting more balanced but less diverse communities compared to the Office (Figure 9).

**FIGURE 9:**
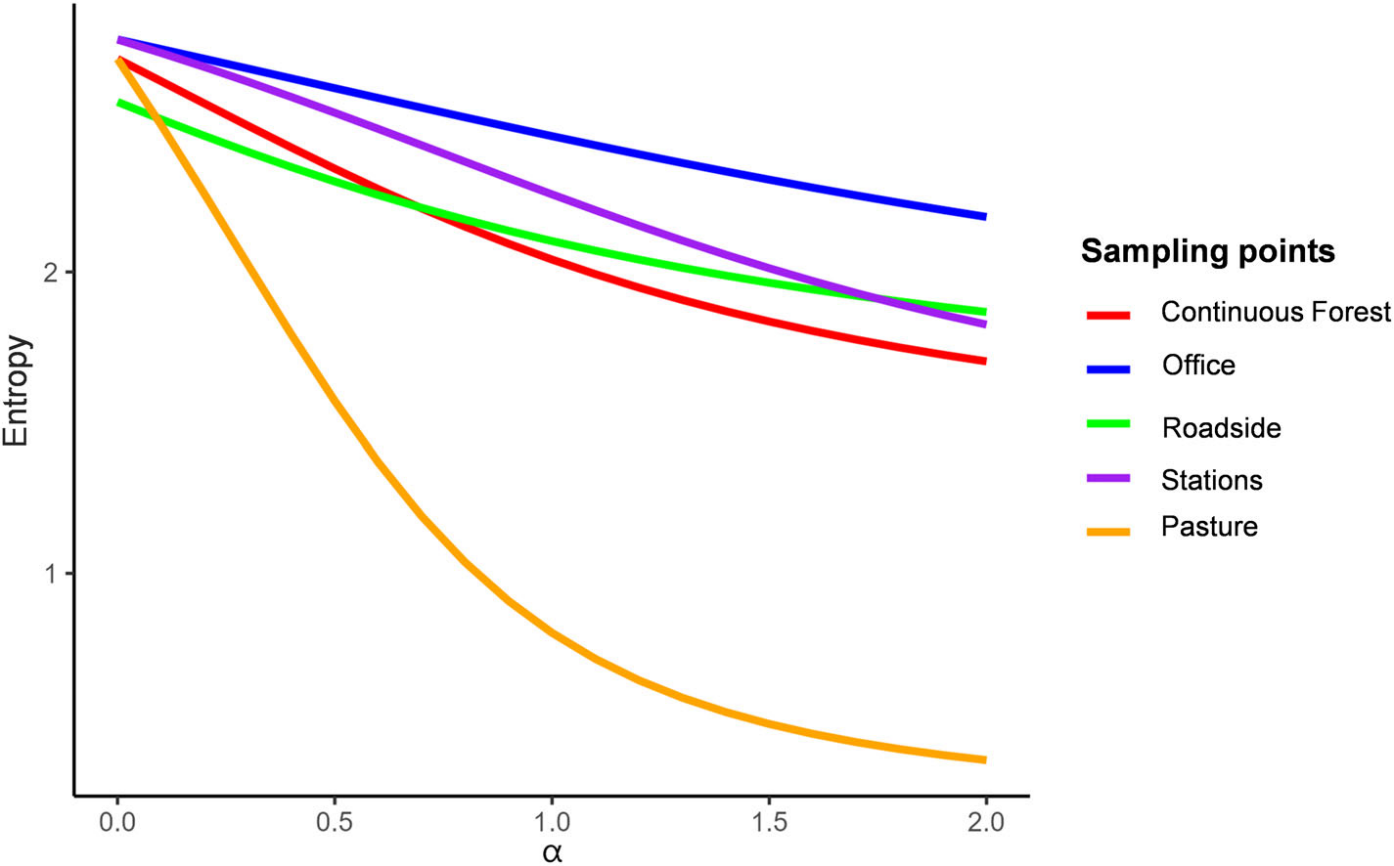
Rényi diversity curves showing differences across the five sampling units: Continuous Forest, Roadside, Office, Stations, and Pasture. *X*‐axis: *α* = 0 (species richness); *α* = 1 (Shannon–Weiner index); *α* = 2 (Simpson–Yule index); *α* = ∞ (Berger–Parker index).

Rarefaction and extrapolation curves revealed that the Office unit exhibited the highest observed and extrapolated species richness, followed by the Roadside unit, which also showed high projected richness. Continuous Forest and Stations displayed intermediate richness with moderate potential for increased diversity. In contrast, the Pasture unit showed the lowest richness, both observed and projected, indicating limited diversity even with increased sampling. Shaded areas representing confidence intervals were wider in locations with greater estimation uncertainty (e.g., Office and Roadside) and narrower in Pasture, indicating higher precision in that environment (Figure 10).

**FIGURE 10:**
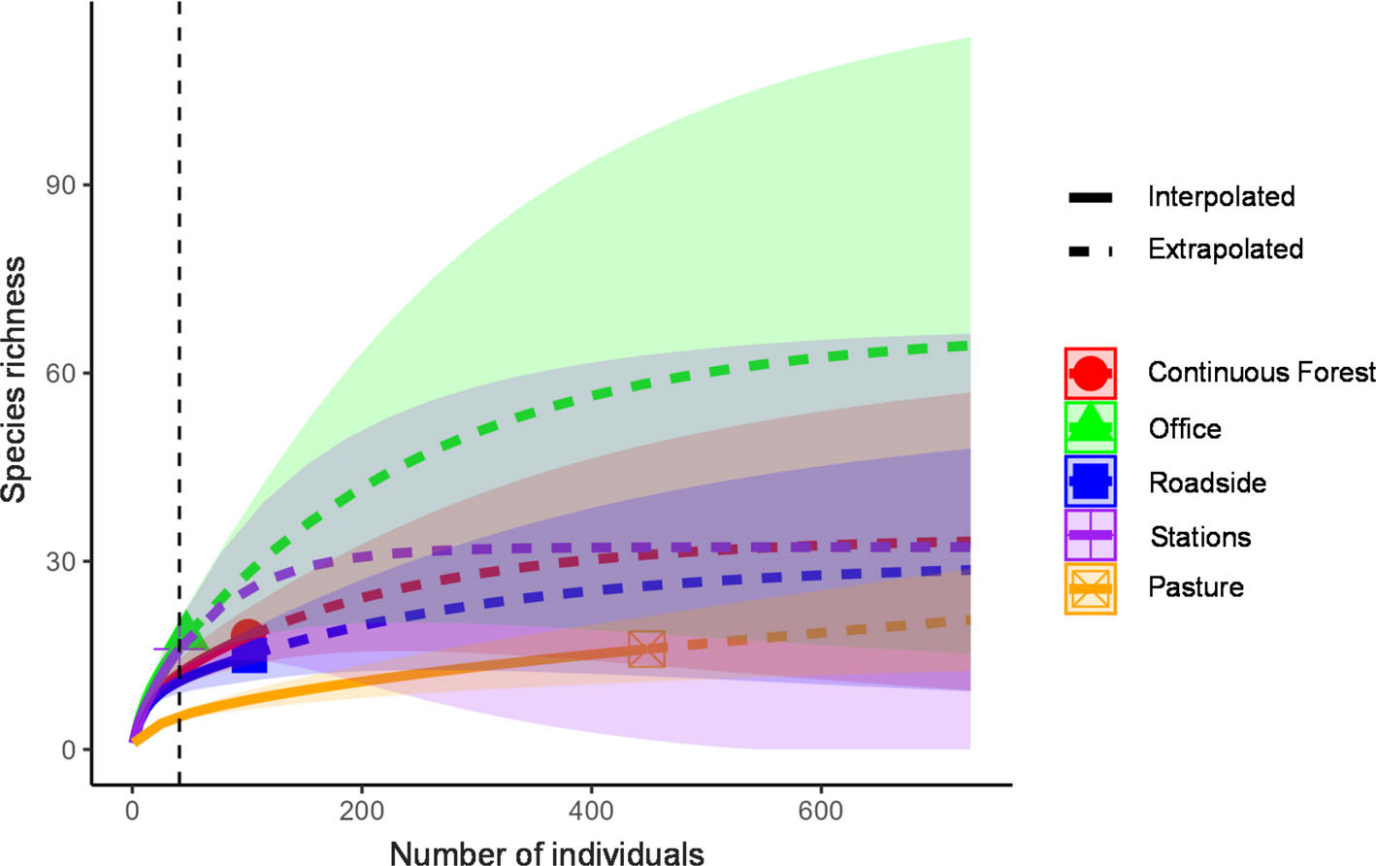
Interpolation and extrapolation curves based on the number of individual specimens collected across five sampling units. Shaded areas represent 95% confidence intervals.

PCoA confirmed dissimilarity in culicid composition among sampling units. The first two axes explained 61.24% of total variation, 49.39% by axis 1 and 11.85% by axis 2. Overlap between clusters indicated shared species among units, though clear separation was noted between Pasture and both Continuous Forest and Stations, associated with the presence of *Aedeomyia squamipennis* (sp.1), *Ae*. *albopictus* (sp.8) and *Cx*. *quinquefasciatus* (sp.16), suggesting distinct community composition. The Office unit occupied a central position on the plot, indicating lower dissimilarity relative to other units. Roadside and Stations showed high overlap, suggesting substantial community similarity (Figure 11).

**FIGURE 11:**
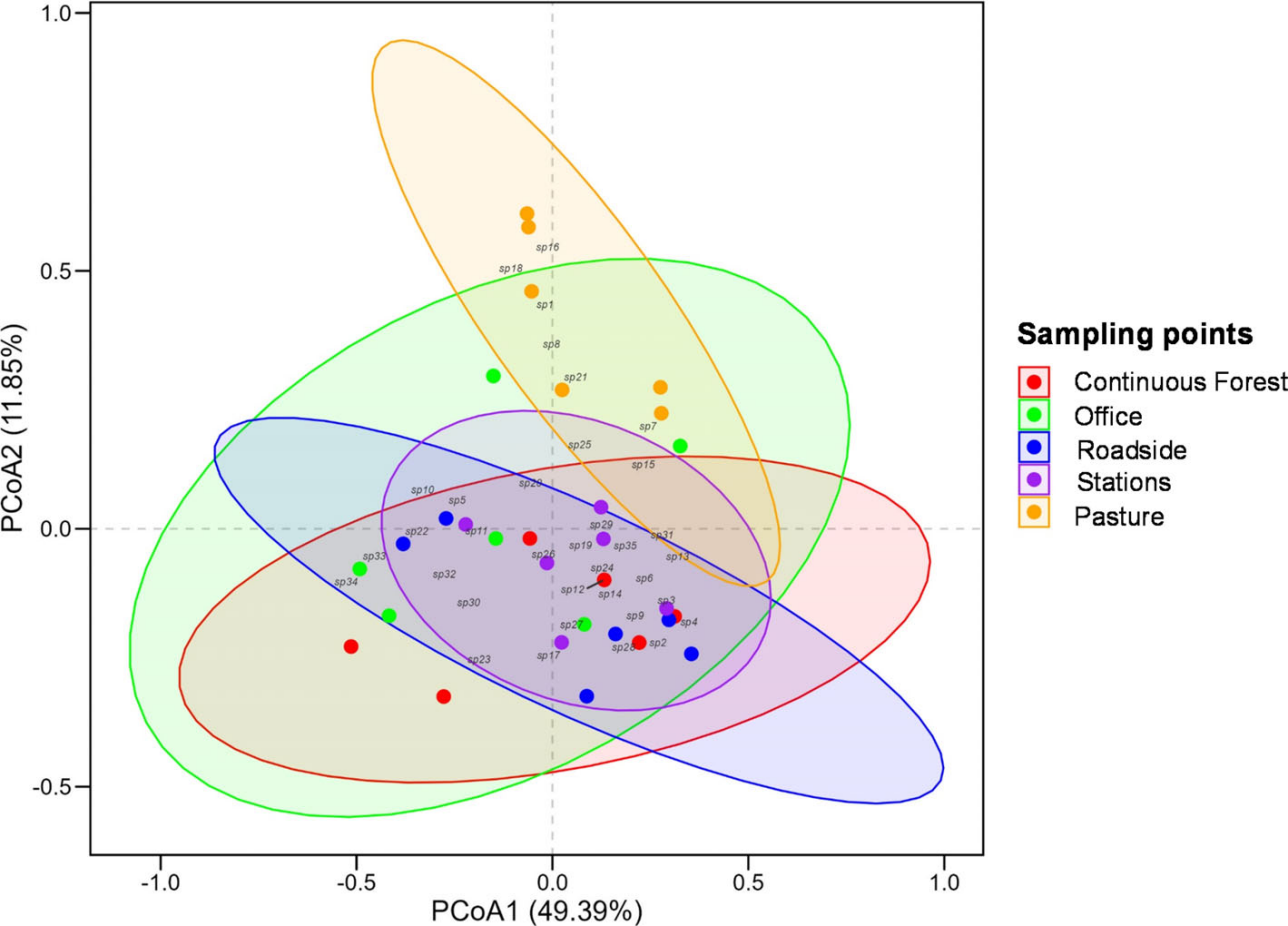
Principal Coordinates Analysis (PCoA) based on Bray–Curtis distances, comparing culicid communities across the five sampling units.

The Venn diagram illustrated species overlap among the five sampling units, while also highlighting the presence of unique taxa at specific sites. The Stations, Pasture and Roadside units showed a notable number of exclusive species. Unique to the Stations were *Haemagogus leucocelaenus*, *Sabethes glaucodaemon* and *Wyeomyia aporonoma*. In the Pasture unit, *Ad*. *squamipennis*, *Aedes argyrothorax* and *Lutzia bigoti* were exclusive. Roadside exclusive species included *Aedes hastatus*, *Mansonia pseudotitilans* and *Psorophora cilipes*. A core group of species, *Ae*. *serratus* and *Ps*. *ferox*, was shared across all sampling units, suggesting high ecological plasticity of these taxa. As observed in the PCoA (Figure 11), the intersections between Continuous Forest and Office, as well as between Roadside and Stations, indicate ecological similarities, reflected by the shared species composition (Figure 12).

**FIGURE 12:**
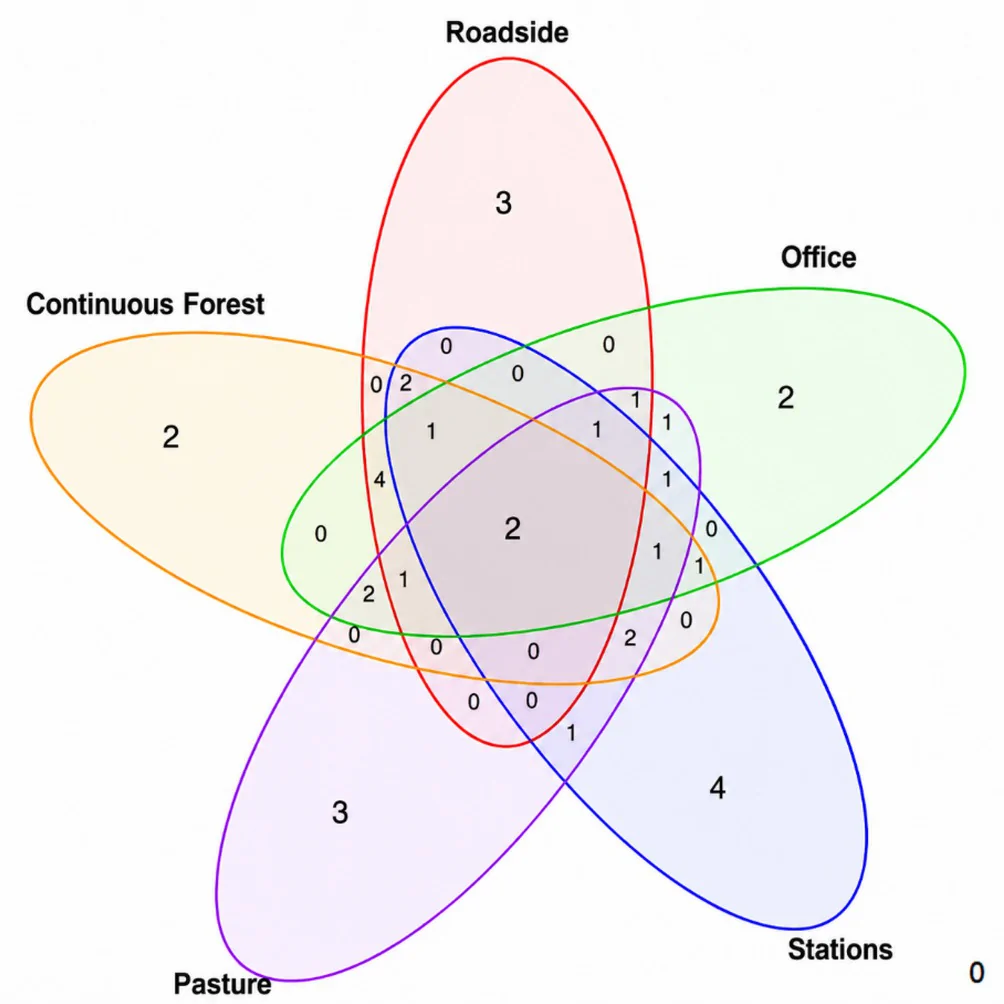
Venn diagram showing unique and shared mosquito species among the five sampling units located in the area of Mineração Paragominas S.A. (MPSA), Paragominas, PA, Brazil.

### Blood‐feeding patterns in mosquitoes

During the study period, 36 blood‐fed mosquitoes were collected, all from the subfamily Culicinae, distributed across five genera and 11 species. Molecular analysis using the 28S rRNA D2 fragment identified species such as *Ps*. *ferox*, *Cx*. *coronator*, *Culex ybarmis* and *Culex urichii* (Table 3).

Nine vertebrate species were identified as blood meal sources, including seven mammals and two reptiles. Four mosquito samples contained multiple blood sources, making exact identification difficult. The most frequently used hosts were humans (*Homo sapiens*, 27.77%), tapir (*Tapirus terrestris*, 19.44%) and red brocket deer (*Mazama americana*, 16.66%) (Table 4).

**TABLE 4:** Composition of blood‐fed mosquito species and identification of their vertebrate blood meal sources, collected in the Mineração Paragominas S.A. (MPSA) area, Paragominas, PA, from July 2022 to May 2023.

Mosquitoes			Host vertebrates	
Genus	Subgenus	Specie	Species Authority, date	No
*Aedes*	(*Ochlerotatus*)	*scapularis*	*Dasyprocta* sp.	1
		*serratus*	*Tapirus terrestris* (Linnaeus, 1758)	1
			*Homo sapiens* Linnaeus, 1758	2
*Psorophora*	(*Grabhamia*)	*dimidiata*	*Mazama americana* (Erxleben, 1777)	1
	(*Janthinosoma*)	*ferox*	*Homo sapiens* Linnaeus, 1758	1
			*Tapirus terrestris* (Linnaeus, 1758)	1
			*Mazama americana* (Erxleben, 1777)	1
*Culex*	(*Carrollia*)	*urichii*	*Anolis punctatus* Duadin, 1802	1
	(*Culex*)	*quinquefasciatus*	*Tapirus terrestris* (Linnaeus, 1758)	2
			*Homo sapiens* Linnaeus, 1758	1
			*Bos taurus* Linnaeus, 1758	1
		*coronator*	*Tapirus terrestris* (Linnaeus, 1758)	1
			*Mazama americana* (Erxleben, 1777)	4
			*Homo sapiens* Linnaeus, 1758	1
			*Cuniculus paca* Linnaeus, 1766	1
			Multiple blood feeding	2
		sp.	*Homo sapiens* Linnaeus, 1758	1
	(*Melanoconion*)	*ybarmis*	*Bos taurus* Linnaeus, 1758	1
			*Sus scrofa* Linnaeus, 1758	1
			*Homo sapiens* Linnaeus, 1758	1
			*Tapirus terrestris* (Linnaeus, 1758)	2
			*Uranoscodon superciliosus* Linnaeus, 1758	1
		sp.	Multiple blood feeding	2
			*Homo sapiens* Linnaeus, 1758	1
	(*Phenacomyia*)	*corniger*	*Homo sapiens* Linnaeus, 1758	1
*Coquillettidia*	(*Rhynchotaenia*)	*arribalzagae*	*Dasyprocta* sp.	1
		*lynch*	*Dasyprocta* sp.	1
*Uranotaenia*		sp.	*Homo sapiens* Linnaeus, 1758	1
			Total number of vertebrates	9
			Total number of mosquitoes	36

Aedini mosquitoes (*Aedes* and *Psorophora*) fed exclusively on mammals (four species), while Culicini (*Culex*) fed on eight vertebrates, including mammals and reptiles. The tribes Mansoniini (*Coquillettidia*) and Uranotaeniini (*Uranotaenia*) each fed on a single vertebrate species (Figure 13).

**FIGURE 13:**
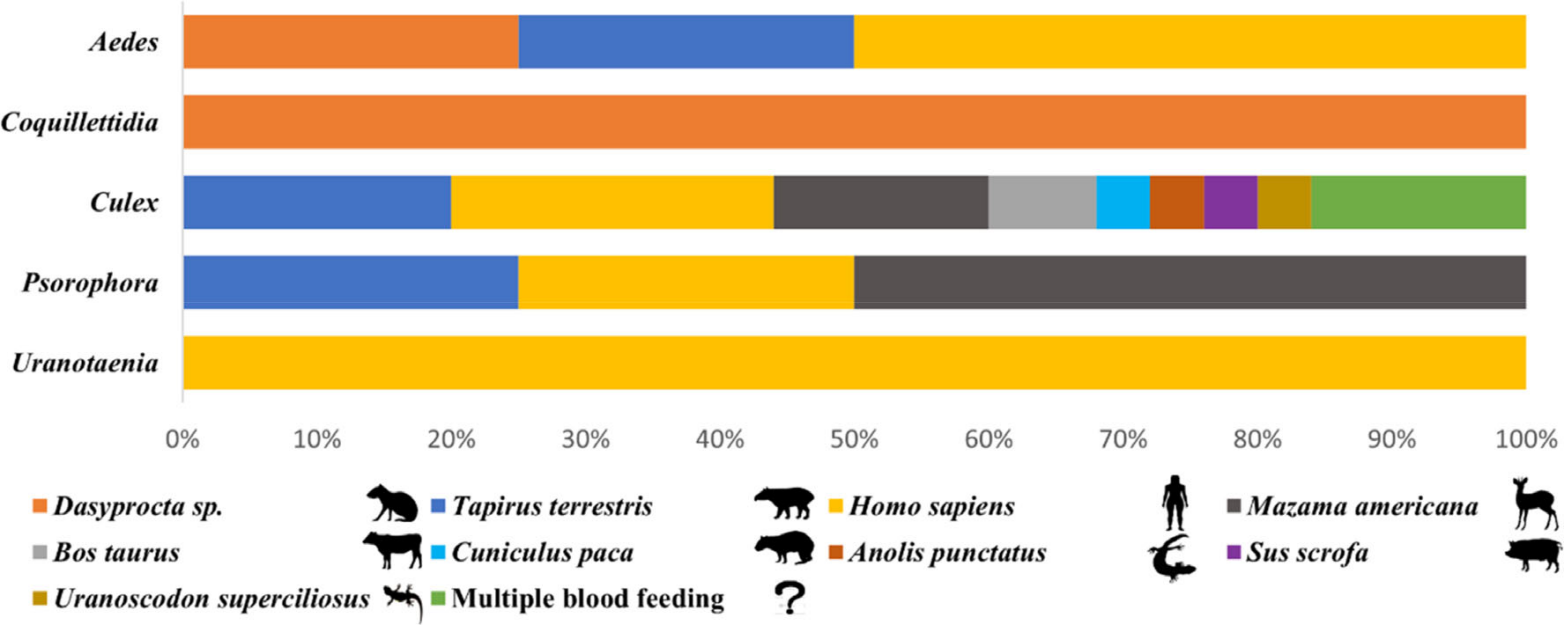
Relative frequency of vertebrate blood sources used by mosquito genera collected in the area of Mineração Paragominas S.A. (MPSA), Paragominas, PA, from July 2022 to May 2023.

### Mining reduces availability of blood sources

In the Forest area, 15 blood‐fed mosquitoes were collected, representing five genera and seven species. The most abundant were *Cx*. *ybarmis* (*n* = 4), *Ae*. *serratus* (*n* = 3) and *Ps*. *ferox* (*n* = 2). These mosquitoes fed on eight different vertebrate species, with one individual showing evidence of multiple blood meals. The most frequent hosts were humans (*n* = 5) and the agouti (*Dasyprocta* sp.; *n* = 3) (Figure 14).

In the Mining area, 21 blood‐fed mosquitoes were collected, belonging to two genera and six species. The most abundant were *Cx*. *coronator* (*n* = 9), *Cx*. *quinquefasciatus* (*n* = 4) and *Culex* (*Melanoconion*) sp. (*n* = 2). These mosquitoes fed on five vertebrate species, with three individuals showing evidence of multiple meals. The most frequent hosts were the tapir (*n* = 6), humans (*n* = 5) and red brocket deer (*n* = 5) (Figure 14).

**FIGURE 14:**
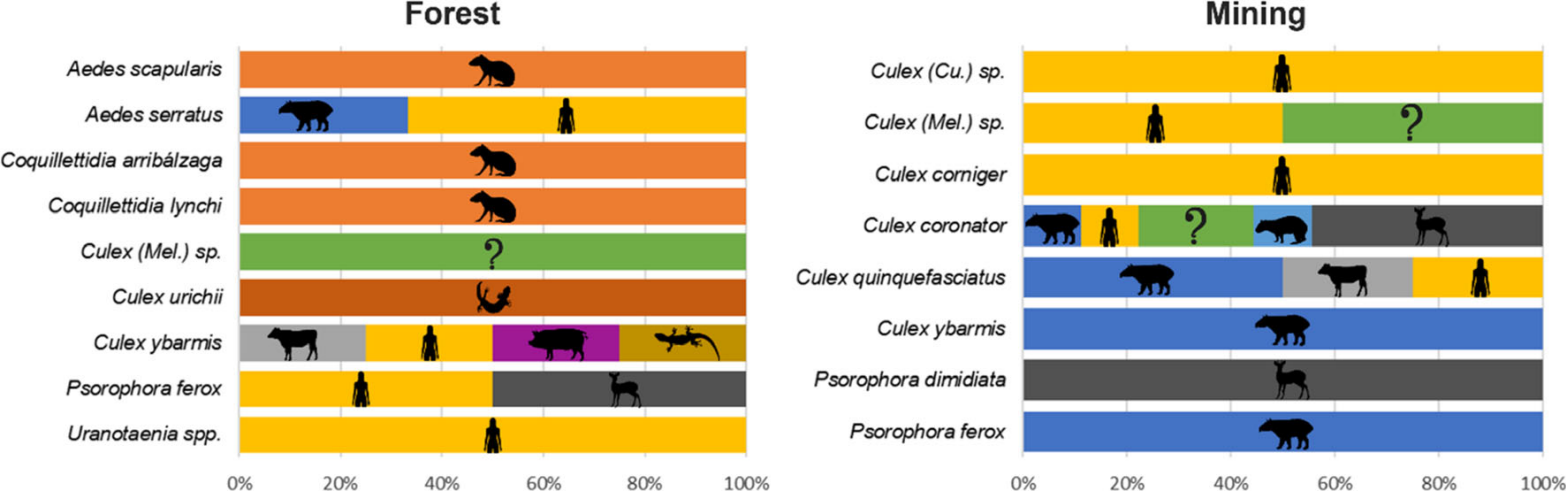
Comparative relative frequency of vertebrate blood sources used by mosquitoes collected in the area of Mineração Paragominas S.A. (MPSA), Paragominas, PA, from July 2022 to May 2023.

### Habitat heterogeneity influences mosquito feeding behaviour

The composition of blood‐fed mosquitoes and the diversity of their blood sources varied markedly among sampling units. In the Continuous Forest, 10 blood‐fed specimens were collected, with *Cx*. *ybarmis* (*n* = 4) being the most frequent, and the agouti (*n* = 3) the most common host. A single instance of multiple feeding was recorded. At the Roadside unit, five blood‐fed mosquitoes were captured, with *Ae*. *serratus* and *Ps*. *ferox* being the most common species (*n* = 2 each) and humans (*n* = 3) representing the most frequently used host (Figure 15).

In the Office unit, four blood‐fed mosquitoes were collected. *Cx*. *coronator* (*n* = 3) was the most abundant species, and human blood was again the most commonly detected (*n* = 2). At the Stations unit, four blood‐fed mosquitoes were also collected, with *Cx*. *coronator* (*n* = 2) being the most frequent species. All blood sources were equally represented (*n* = 1), and one individual showed multiple blood meals. In the Pasture unit, 13 blood‐fed mosquitoes were collected, with *Cx*. *coronator* and *Cx*. *quinquefasciatus* being the most frequent species (*n* = 4 each). The tapir (*n* = 5) was the main blood source, and two cases of multiple feeding were observed (Figure 15).

**FIGURE 15:**
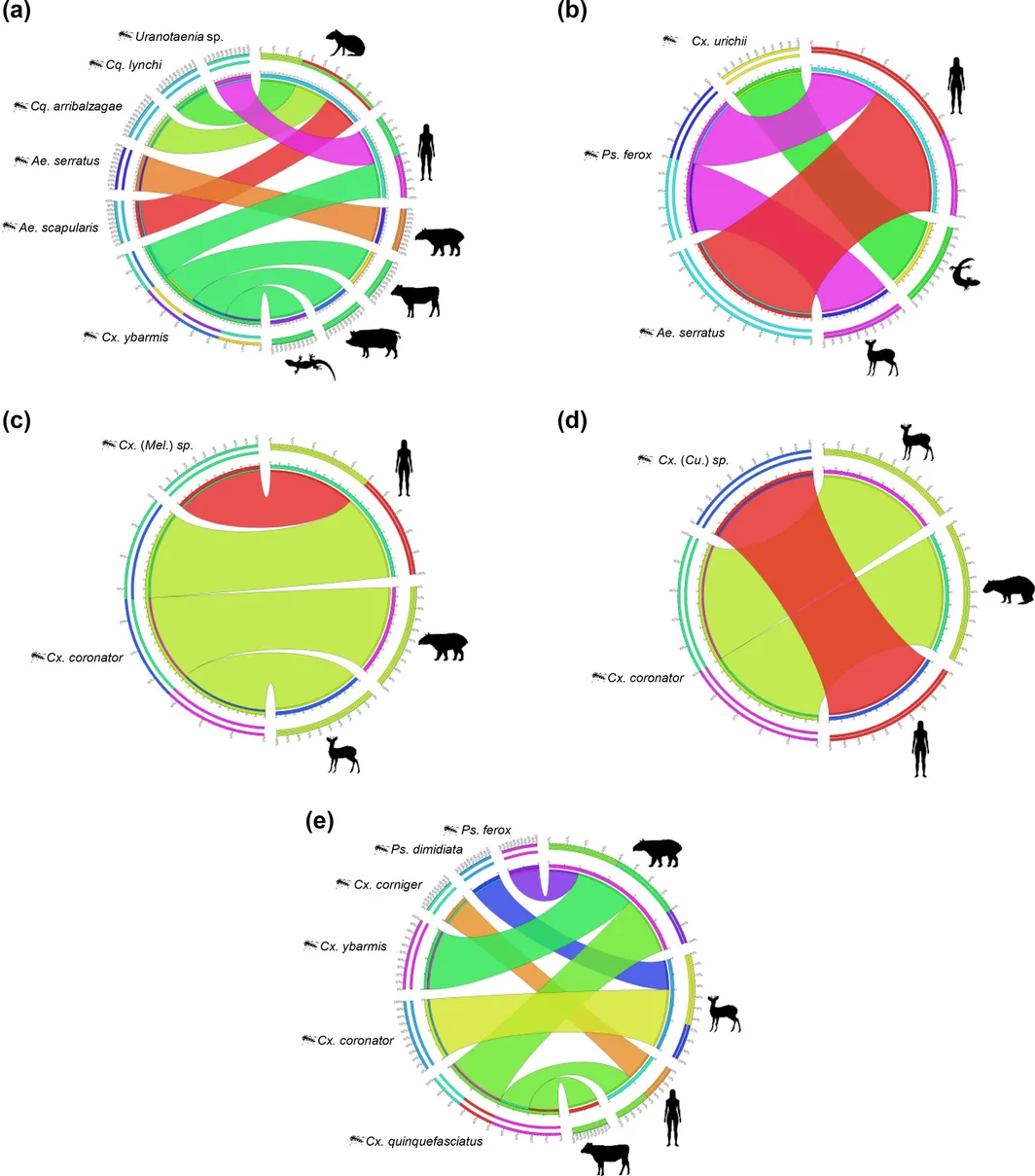
Circos plots showing mosquito species and their blood‐feeding sources. (a) Continuous Forest: One multiple blood meal by *Cx*. (*Mel*.) sp.; (b) Roadside: Mosquito–host interactions and frequencies; (c) Office: Mosquito–host interactions and frequencies; (d) Stations: One multiple blood meal by *Cx*. (*Mel*.) sp.; (e) Pasture: Two multiple blood meals by *Cx*. *coronator*.

## DISCUSSION

This study confirmed the hypothesis that mosquito diversity, community composition and blood‐feeding patterns are influenced by bauxite mining activities. These changes reflect the environmental heterogeneity of each site, shaped by factors such as vegetation cover, larval habitat availability, shading, ecological integrity, abundance of floral and blood resources, microclimatic conditions and the level of anthropogenic impact (Chaves et al., 2011; Hendy et al., 2025; Lovett et al., 2005).

Although differences in species diversity among sampling units were subtle, significant changes were observed in community composition, similar to findings in previous studies (Hendy et al., 2020, 2025; Meyer Steiger et al., 2016; Orlandin et al., 2020; Young et al., 2021). These changes are often associated with the influx of generalist species adapted to open or edge habitats and habitat heterogeneity driven by human activity. Our results support this pattern, revealing increased species richness and abundance, particularly of vector and anthropophilic species, along mining edges. These findings reinforce the view that changes in mosquito community composition are linked to such environmental factors.

The observed increase in mosquito richness and abundance in mining areas may be explained by species overlap, where synanthropic and anthropophilic species coexist with forest specialists. This pattern is common in dense vegetation areas that are suddenly altered by human activity, leading forest species to settle in edge habitats that appear favourable due to higher microhabitat diversity and availability of blood sources. However, these environments tend to favour anthropophilic species with competitive advantages, displacing forest specialists over time (Fahrig, 2017; Kark & Rensburg, 2006; Moi et al., 2020). This phenomenon aligns with the concept of an ecological trap, where abrupt environmental changes alter settlement cues, increasing site attractiveness while reducing its suitability for survival and reproduction (Robertson & Hutto, 2006).

Another key finding was that mosquito community composition was sensitive to the degree of environmental disturbance. According to Junglen et al. (2009), *Aedes*, *Anopheles* and *Culex* are commonly associated with anthropogenic environments and are more frequently implicated in virus isolations, including members of the Bunyaviridae, Coronaviridae, Flaviviridae and Rhabdoviridae families. In contrast, *Uranotaenia*, typically found in primary forests, is associated with fewer viral isolations. This distinction was evident in our study: while *Culex*, *Aedes*, *Psorophora*, and *Uranotaenia* dominated the Forest area, the Mining area was dominated by *Culex*, *Aedes*, *Nyssorhynchus* and *Psorophora*. Orlandin et al. (2020) support the idea that the abundance of specific taxa may serve as indicators of habitat disturbance.

Among the recorded genera, the predominance of *Culex* across all sampling units reflects its broad adaptability to diverse environmental conditions (Mayi et al., 2019; Srisuka et al., 2022). This high abundance has also been reported in other studies conducted in Pará (Confalonieri & Costa Neto, 2012; Mondet et al., 2002; Nunes et al., 2009; Reis et al., 2025; Santos et al., 2005) and in other Amazonian regions (Hutchings et al., 2016, 2018, 2020). Moreover, *Culex* dominance in many surveys may be linked to the use of CDC light traps, typically operated for 12 h at night, which favour collection of crepuscular and nocturnal species, particularly from the subfamily Anophelinae, Culicini and Uranotaeniini tribes of Culicinae (Consoli & Oliveira, 1994; Forattini, 2002). These three groups accounted for over 83% of all specimens collected in this study.

*Culex quinquefasciatus* was the most abundant species in the study, recorded in nearly all mining units. Its presence in forest edges is noteworthy due to its synanthropic and anthropophilic behaviour (Medeiros‐Sousa et al., 2017; Wilke et al., 2019). Most individuals were collected in the Pasture unit, an early post‐mining regeneration site containing nursery plots, administrative buildings and a septic tank near the forest. These features provide ideal conditions for colonisation and proliferation of *Cx*. *quinquefasciatus*, particularly in ecologically recovering areas with unoccupied niches.

The species is known for its ecological plasticity and well adapted to anthropogenic environments; it lays eggs in a wide range of larval habitats (e.g., septic tanks) and has a high reproductive rate (Forattini, 2002; Medeiros‐Sousa et al., 2017; Wilke et al., 2019). The predominance of males observed in Pasture, frequently found near breeding sites, further supports the association between species abundance and artificial larval habitat availability. Our findings highlight the remarkable dispersal and adaptation capabilities of *Cx*. *quinquefasciatus*, even in remote Amazonian regions (Chapman et al., 2015; Lühken et al., 2023), as well as the potential public health risk it poses due to its role as a vector of multiple pathogens (Harvey‐Samuel et al., 2021; LaPointe, 2012).

Other medically important *Culex* species were also abundant in the Mining area, including *Cx*. *coronator*, *Cx*. *corniger* and species from the *Melanoconion* subgenus. These mosquitoes are broadly distributed, occurring in both forested and urban environments, and use a wide range of breeding sites (Forattini, 2002). Their higher abundance in the Mining area suggests increased adaptation and reproductive success under anthropogenic disturbance. Both *Cx*. *coronator* and especially *Melanoconion* species are recognised vectors involved in enzootic cycles of various arboviruses (Alto et al., 2014; Segura & Castro, 2007; Shope et al., 1964). *Melanoconion* mosquitoes, collected mainly in Continuous Forest and Pasture, are known vectors of viruses historically isolated in Pará, such as Guamá virus (GMAV), Moju virus (MOJUV), Ananindeua virus (ANUV), among others (Causey et al., 1961; Mitchell et al., 1985; Shope et al., 1988).

The genus *Aedes* was the second most abundant in this study, a pattern also observed in other parts of the Amazon (Hutchings et al., 2016, 2018, 2020; Saraiva et al., 2020), although in other regions its presence has been less prominent (Confalonieri & Costa Neto, 2012; Santos et al., 2005). *Aedes serratus*, *Ae*. *scapularis* and *Ae*. *arborealis* were predominant in the Forest area, while *Ae*. *albopictus* was found exclusively in the Mining area, consistent with studies reporting its greater abundance along forest edges near anthropized zones, with reduced presence deeper into vegetation (Hawley, 1988; Hendy et al., 2020). Although *Ae*. *arborealis* has no confirmed vector role, several studies have linked *Ae. serratus*, *Ae*. *scapularis* and *Ae*. *albopictus* to the transmission of arboviruses (Cardoso et al., 2010; Garcia‐Rejon et al., 2021; Iversson, 1980). *Aedes albopictus*, in particular, is susceptible to at least 26 arboviruses, some with pandemic potential (Paupy et al., 2009). Despite confirmed vector competence and reports of natural infections in Brazil (Ferreira‐de‐Lima & Lima‐Camara, 2018; Honório et al., 2018), the species has not been implicated as a primary vector for major arboviruses like DENV, CHIKV or ZIKV.

*Psorophora* was the third most abundant genus in this study, similar to findings from the Padauari and Araçá Rivers in Amazonas state (Hutchings et al., 2016, 2018), though in other regions, such as Combu Island (Pará), it was even the most dominant group (Farias, 2019). The most frequently encountered species were *Ps*. *ferox* and *Ps*. *dimidiata*, which displayed distinct distributions: *Ps*. *ferox* dominated the Forest area, while *Ps*. *dimidiata* was more abundant in the Mining area, a result consistent with their known ecological preferences (Forattini, 2002; Stein et al., 2011, 2022). *Psorophora ferox* is particularly notable for its vector potential, having been found naturally infected with Saint Louis encephalitis virus (SLEV), West Nile virus (WNV), Ilheus virus (ILHV) and Rocio virus (ROCV) (Causey et al., 1961; Lopes et al., 1978; Maia et al., 2024).

Although less dominant, other species also stood out for their abundance or epidemiological relevance, especially in forest areas. *Uranotaenia calosomata* and *Or*. *fascipes* were particularly abundant in the Forest. *Uranotaenia calosomata* is considered a forest specialist, breeding in natural habitats such as riverbanks, rock holes and phytotelmata, and is sensitive to environmental disturbance (Orta‐Pineda et al., 2021; Srisuka et al., 2022). It feeds primarily on amphibians and reptiles but may also feed on birds and mammals, with no current evidence of pathogen transmission. *Orthopodomyia fascipes*, typically found in phytotelmata and rarely in artificial habitats, has ornithophilic behaviour and is suspected to be involved in avian arbovirus cycles, although its bioecology and vector competence remain poorly understood (Forattini, 2002; Hanson et al., 1995).

Additional medically relevant species were also recorded, though in low numbers. *Aedes fulvus*, primarily collected in the Forest, has been found naturally infected with yellow fever virus (YFV) and Venezuelan equine encephalitis virus (VEEV) (Vasconcelos, 2003). In the Mining area, *Hg. leucocelaenus* and *Sa. glaucodaemon* were found exclusively at the Stations unit. Typically associated with canopy strata, their ground‐level collection may indicate vertical structure disruption in edge habitats (Hendy et al., 2020, 2025), or behaviour shifts towards ground‐level host‐seeking or oviposition. *Haemagogus leucocelaenus*, and possibly *Sa*. *glaucodaemon*, are important YFV vectors in sylvatic cycles (Abreu et al., 2019; Caleiro et al., 2025; Vasconcelos, 2003).

Other noteworthy species include *Coquillettidia venezuelensis*, more frequent in the Mining area and previously found naturally infected with Mayaro virus (MAYV), Oropouche virus (OROV) and SLEV (Aitken et al., 1969; Anderson et al., 1961; Hervé et al., 1986); and *Trichoprosopon digitatum*, collected in the Forest, with documented infections by Pixuna virus (PIXV), Wyeomyia virus (WYOV), SLEV and ILHV (Causey et al., 1961; Galindo et al., 1966; Shope et al., 1964). These findings highlight the diversity of potential vector species, even at low densities and underscore the importance of ongoing surveillance in diverse environments.

This study provided a broad collection of mosquito genera in the state of Pará, with the exception of the genera *Chagasia* (Xavier & Mattos, 1975) and *Johnbelkinia* (Nunes et al., 2009). Additionally, we report the occurrence of *Cx*. *ybarmis* and *Ps*. *dimidiata* in Pará, two species not previously documented in surveys of the state (Confalonieri & Costa Neto, 2012; De Araújo et al., 2020; Dias, 2023; Farias, 2019; Mondet et al., 2002; Moy et al., 2024; Nunes et al., 2009; Pinheiro et al., 1981; Reis et al., 2025; Ribeiro, 2018; Santos et al., 2005; Segura et al., 2003). However, their roles in pathogen transmission remain unclear; what is known is that both species exhibit broad host preferences and aggressive biting behaviour, especially *Ps*. *dimidiata* (Soghigian et al., 2023; Stein et al., 2011, 2022).

Blood meal analyses revealed high vertebrate diversity, with important implications for pathogen transmission in the mining region. The genus *Culex* showed remarkable feeding plasticity, utilising eight different vertebrate hosts, including humans, tapirs, deer, pacas and lizards, reinforcing its role as a potential bridge vector in arbovirus transmission, including WNV (Soghigian et al., 2023). Several of these hosts are known reservoirs of arboviruses such as bluetongue virus (BTV), eastern equine encephalitis virus (EEEV), VEEV, ZIKV and others (Bosco‐Lauth et al., 2018; de Kesel et al., 2024; Medici et al., 2014; Reeves & Burkett‐Cadena, 2022; Santos et al., 2024).

In contrast, *Aedes* and *Psorophora* were more specialised, feeding exclusively on mammals, a trait shaped by their evolutionary histories and feeding adaptations (Rose et al., 2020; Soghigian et al., 2023). Humans were the most common blood source, followed by tapirs, brocket deer and agoutis, wild rodents that inhabit core forest areas and are involved in the maintenance of enzootic cycles (de Kesel et al., 2024; dos Santos et al., 2009), all of which are recognised reservoirs for multiple arboviruses (de Kesel et al., 2024; Medici et al., 2014; Santos et al., 2024). Although the genus *Coquillettidia* is also known for high feeding plasticity (Soghigian et al., 2023), in this study, it showed an exclusive association with agoutis.

Blood meal analyses showed that the Mining area exhibited lower richness of both engorged mosquitoes and vertebrate hosts. This reduction undermines the dilution effect typical of preserved ecosystems, in which high vertebrate diversity reduces the likelihood of pathogen transmission (Keesing et al., 2006; LoGiudice et al., 2003). The situation is aggravated by the dominance of efficient vector species in the mining landscape, and when coupled with reduced host availability, this increases mosquito–human contact and exposure to pathogen reservoirs. In contrast, the Forest area maintained high richness of both blood‐fed mosquitoes and vertebrate hosts, preserving the dilution effect even in the presence of competent vectors, and thereby reducing spillover risk (Ostfeld & Keesing, 2012).

Nonetheless, the identification of humans as the main blood source in the Forest area is surprising and concerning. Possible explanations include occupational exposure of field collectors and mining workers, or illegal hunting activity, both common in the Amazon region (Antunes et al., 2019). Notably, the detection of *Sus scrofa* blood meals in the Continuous Forest unit, located 3 km from animal enclosures and 2.4 km from a forest‐farm edge, suggests two mechanisms: (1) *Culex* mosquitoes, known for long‐distance flight capacity including high‐altitude wind‐assisted dispersal (Atieli et al., 2023), can explore long areas and/or (2) domestic animals may traverse the entire farm property or escape enclosures, entering forest edges where they encounter sylvatic mosquito populations. These findings demonstrate how vector mobility and host movements can connect isolated ecosystems. This behaviour underscores their capacity to bridge sylvatic and peri‐domestic transmission cycles, amplifying spillover and spillback risks (Hendy et al., 2023; Vieira et al., 2022). Similarly, the prominence of tapir and brocket deer as blood sources in the Mining area likely reflects their abundance and tolerance to environmental degradation (Teixeira‐Santos et al., 2020), potentially facilitated by protection from large predators within the mining area and the availability of food resources in reforested zones (Cerqueira et al., 2021; Fragoso, 1997; Novack et al., 2005; Trolle et al., 2008).

These interspecific interactions, combined with environmental changes driven by mining, suggest that the mining area functions as a hotspot for vector–pathogen–host interactions. Given the complexity of transmission cycles, which involve the interplay of vectors, pathogens and hosts under strong environmental pressures (Huang et al., 2019; Swei et al., 2020), the probability of cross‐species transmission is high, especially considering the high mutation rates of arboviruses, most of which are RNA viruses. These characteristics enhance their capacity to adapt to novel vertebrate and mosquito hosts (Coffey et al., 2013; Swei et al., 2020; Xiao et al., 2025).

Although this study contributes to the understanding of mosquito communities in mining areas, certain limitations must be acknowledged. The primary challenge was species‐level identification, which was hindered by damage to key morphological characters, likely due to dense vegetation, specimen age and collection/storage conditions. In addition, the predominance of female *Culex*, a genus known for its identification difficulties due to limited diagnostic features, restricted finer‐scale analyses. Still, the taxonomic resolution achieved was sufficient to detect significant shifts in community composition, demonstrating the robustness of the observed ecological patterns.

Another limitation was the exclusive use of terrestrial sampling methods (active search and CDC traps), which may have underestimated canopy‐dwelling species, although a few were captured incidentally. Incorporating complementary techniques such as flight interception traps (Malaise, Shannon) and larval surveys would likely expand species representation, as shown in other studies (Hutchings et al., 2016, 2018, 2020). Moreover, the spatial coverage of the study was constrained by operational limitations in mining environments, including access distance between sites and limited personnel, which may have affected spatial representativeness.

Future studies should expand the sampling area to better capture environmental heterogeneity within both mining and forest landscapes. A multi‐method approach encompassing ground, canopy and larval sampling would improve species coverage. Furthermore, integrating morphological and molecular identification tools is essential to overcome species‐level diagnostic challenges, especially for critical groups like *Culex*. Research focusing on edge gradients could also clarify how microclimatic factors (e.g., temperature, humidity) influence mosquito distribution. Further, the sample size of blood‐fed mosquitoes should be increased to get a better resolution in blood‐meal sources. Lastly, implementing integrated surveillance programs, monitoring vectors, pathogens and wildlife hosts, will be key to anticipating pathogen emergence in extractive landscapes and developing more effective, ecosystem‐adapted control strategies (Catenacci et al., 2018).

## CONCLUSION

This study reveals that bauxite mining in the Eastern Amazon significantly alters culicid community structure, favouring generalist and anthropophilic vectors such as *Ae. albopictus* and *Cx. quinquefasciatus* in edge habitats, while reducing overall species and blood source diversity. Molecular analyses identified humans, tapirs and deer as dominant blood sources, with lower vertebrate richness in mining areas, thereby intensifying pathogen transmission risks due to the loss of the dilution effect. These findings highlight mining areas as potential hotspots for spillover and spillback events, underscoring the need for integrated monitoring (vectors–pathogens–hosts) and artificial breeding site control to mitigate public health threats.

## AUTHOR CONTRIBUTIONS

**Mayron Mielly Morais Pacheco:** Conceptualization; writing – original draft; methodology; investigation; writing – review and editing; data curation. **Poliana da Silva Lemos:** Supervision; writing – review and editing; project administration; methodology; conceptualization; investigation; data curation. **Mônica Silva Coelho:** Methodology; investigation; writing – review and editing; data curation. **Tatiane Marques Porangaba de Oliveira:** Methodology; data curation; writing – review and editing. **Luciano Chaves Franco Filho:** Investigation; software. **Øystein Wiig:** Investigation; funding acquisition; writing – review and editing. **Ana Cristina Mendes‐Oliveira:** Funding acquisition; investigation; writing – review and editing. **Leonardo Sena:** Project administration; supervision; funding acquisition; writing – review and editing; formal analysis; data curation. **Maria Anice Mureb Sallum:** Supervision; writing – review and editing; project administration; methodology; conceptualization; data curation; validation; formal analysis; funding acquisition.

## FUNDING INFORMATION

This research is part of the project “Metagenomic and metabarcoding as a tool for developing One Health in Hydro Area, Paragominas, Pará, Brazil” (4600007977 Hydro/4180 Fadesp), financed by Norsk Hydro through the BRC. The BRC provided a master's degree scholarship to Mayron Mielly Morais Pacheco and postdoctoral scholarships to Poliana da Silva Lemos and Mônica Silva Coelho. The Article Processing Charge (APC) for the publication of this study was funded by the Coordination of Higher Education Personnel Improvement, CAPES (ROR identifier: 00x0ma614). For open access purposes, the authors have applied a Creative Commons CC BY licence to any accepted version of the manuscript.

## CONFLICT OF INTEREST STATEMENT

The authors declare no conflicts of interest.

## Data Availability

The data that support the findings of this study are openly available in Dryad at https://doi.org/10.5061/dryad.k6djh9wm3.
